# Effects of verbal tasks with varying difficulty on real-time respiratory airflow during speech generation in healthy young adults

**DOI:** 10.3389/fpsyg.2023.1150354

**Published:** 2023-06-15

**Authors:** Malin Gullsvåg, Claudia Rodríguez-Aranda

**Affiliations:** Department of Psychology, UiT the Arctic University of Norway, Tromsø, Norway

**Keywords:** verbal tasks, speech breathing, task difficulty, peak expiratory airflow, respiratory function, verbal fluency tasks

## Abstract

**Objective:**

Respiratory function is linked to sensory, affective, and cognitive processes and it is affected by environmental constraints such as cognitive demands. It is suggested that specific cognitive processes, such as working memory or executive functioning, may impact breathing. In turn, various lines of research have suggested a link between peak expiratory airflow (PEF) and cognitive function. However, there is scarce experimental support to the above assertions, especially regarding spoken language. Therefore, the present investigation aims to evaluate whether breathing varies as a function of performing verbal naming tasks with different difficulty levels.

**Methods:**

Thirty healthy young adults, (age *M* = 25.37 years), participated in the study. Participants were required to perform aloud five verbal tasks ranged in order of difficulty: Reading single words, reading a text passage, object naming, semantic and phonemic fluency. A pneumotachograph mask was employed to acquire simultaneously the verbal responses, and three airflow parameters: Duration, peak, and volume at both stages of the respiratory cycle (i.e., inspiration/expiration). Data were analyzed with one-way repeated measures MANOVA.

**Results:**

No significant differences were found between reading single words and object naming. In comparison, distinctive airflow requirements were found for reading a text passage, which were proportionally related to number of pronounced words. Though, the main finding of the study concerns the data on verbal fluency tasks, which not only entailed higher inhaled airflow resources but also a significant PEF.

**Conclusion:**

Our data demonstrated that the most difficult tasks, namely semantic and phonemic verbal fluencies, relying on semantic search, executive function, and fast lexical retrieval of words were those requiring important amount of inhaled airflow and displaying a high peak expiratory airflow. The present findings demonstrated for the first time a direct association between complex verbal tasks and PEF. Inconclusive data related to object naming and reading single words are discussed in light of the methodological challenges inherent to the assessment of speech breathing and cognition in this line of investigation.

## 1. Introduction

Increasing interest has arisen in neuroscientific research related to the interaction between the respiratory system and cognition. Cumulated findings point to a strong association between respiratory mechanisms and cognitive abilities (Morton and Braakhuis, 2021), emotion (Homma and Masaoka, 2008), and physiological functioning (Russo et al., 2017). However, there is still a gap in our understanding concerning how respiratory function is interrelated to the above-mentioned processes. In fact, a current review on the topic (Boyadzhieva and Kayhan, 2021) emphasizes the need to further deepen into the mechanisms underlying which factors and contexts modulate the interaction between breath and specific bodily functions, including cognition. One intriguing issue that is still unsettled is the interaction of cognitive processes and respiration. The main question is whether type of cognitive task affects differentially breathing patterns. The issue of type of task is not a simple one as it interrelates with at least three topics: (a) differences related to the nature of the cognitive operations involved, (b) degree of difficulty, and (c) differences in the way of emitting a cognitive answer. Regarding the first aspect, some studies have investigated how respiratory function relates to a wide-range type of tasks including mental arithmetic, memory, reasoning, attentional tasks, and multitasking (Grassmann et al., 2016). The use of such diverse cognitive tasks is not entirely justified, though there is a tacit implication that specific respiratory patterns can be related to concrete cognitive processes. Unfortunately, the number of studies addressing this topic is scarce, and so far, it is unknown whether specific cognitive abilities are associated with specific breathing demands. Notwithstanding, the issue of task difficulty has been better addressed in the literature and existing data have proved that disregarding the cognitive operations evaluated, higher cognitive load (i.e., higher level of difficulty) increases the speed of respiration when compared to baseline conditions (Grassmann et al., 2016). Of particular interest is that very few of the existent studies have included tasks requiring a verbal response, which brings us to the matter of type of answer required when assessing the respiratory-cognition association. A reason for excluding tasks relying on verbal responses is to avoid the effects of speech generation on the evaluated task. Such an approach is parsimonious, as it becomes possible to understand how different silent cognitive operations influence respiratory measurements without mixing the effects of voicing. Nevertheless, by avoiding tasks with an oral answer, an important range of psychological measures are excluded together with the possibility to understand the relationship between cognitive demands and breathing in tasks relying on an oral response.

It should be noted that the interest in understanding the cognition-breathing relationship during on-going speech has been addressed in linguistic research. In this field of study, the vital role of respiration in the physiology of speech has been acknowledged for nearly a century (Fuchs and Rochet-Capellan, 2021). In spite of it, the link between definite cognitive demands and breathing during speech remains unclear. Previous research has demonstrated that speech generation entails strong adaptations in breathing patterns related to linguistic and cognitive requirements (Serre et al., 2021). For instance, important differences in respiratory outcomes have been reported in healthy individuals required to read short text passages versus executing spontaneous speech during free talking (Winkworth et al., 1994, 1995; Mitchell et al., 1996; Wang et al., 2010). The main differences found by comparing these two conditions were related to longer breath durations, more inappropriate location of inhalations, and more variable patterns of breathing during spontaneous speech (Winkworth et al., 1995). The latter results suggest that spontaneous speech is a demanding activity entailing higher cognitive demands than reading (Wang et al., 2010).

Apart from studies evaluating reading and spontaneous speech, there is scarce empirical data of how additional spoken verbal tasks affect respiratory outcomes. Thus, in order to advance this line of research, it would be convenient to employ tasks restricted to verbal production that elicit particular cognitive processes, such as those used in neuropsychological assessment. In fact, an earlier study by Cahana-Amitay et al. (2018) already applied naming tests and sentence comprehension tests to investigate the association between language performance and pulmonary function in healthy older participants. Findings from this investigation showed that individuals with higher pulmonary function were those with best performance in the language tasks, especially in the Boston naming test, which is highly demanding in terms of visuo-perceptual abilities, semantic memory retrieval, as well as phonological and motor processing (Price et al., 2005). Thus, the authors concluded that object naming imposes higher cognitive demands and requires more brain oxygenation, which in turn causes upregulation of the respiratory function. Such an observation agrees with the proposal that higher mental effort entails higher energy expenditure, which translates among other processes into higher variability of gas exchange parameters during respiration (Grassmann et al., 2016).

The study of Cahana-Amitay et al. (2018) is a timely attempt to further explore the relationship between specific cognitive demands in different oral tasks and respiratory measures (i.e., vital capacity). However, this investigation relied on a correlational approach where cognitive outcomes and respiratory parameters were measured independently. It is actually the case, that most of the studies addressing the relationship cognition-respiratory function for different purposes have relied on correlational designs where the measurements for cognition and respiration are taken separately (e.g., Anstey et al., 2004; Duggan et al., 2019). Therefore, to better understand the effects of type of task on breathing patterns, a reasonable step to follow is to conduct a direct assessment of respiratory measures during speech generation of verbal tasks. However, this attempt is not without complications. The unique characteristic of breathing in adapting to environmental constraints and being influenced by the contexts sets a challenge to the experimental design. Earlier studies assessing respiration during reading vs. spontaneous speech have used a plethora of instrumentations to measure respiration, ranging from inductance plethysmography, video recording, and modern pneumotachographs (Rothenberg mask) (e.g., Wang et al., 2010; Wlodarczak and Heldner, 2020). In addition, other issues arise due to the type of verbal tasks employed. The main difficulty in comparing different verbal tasks is the fact that speech production tends to be highly variable and unpredictable. Therefore, tasks restricting to some degree the verbal output, such as the naming test used by Cahana-Amitay et al. (2018) are appropriate. For this reason, we selected five naming tests with different difficulty level to evaluate their effects on respiration during exact time of speech generation.

## 2. Rationale for degree of difficulty and selection of verbal tasks

We deem necessary to begin this section by drawing attention to the issue of how to define difficulty level in verbal tests. Generally, there is no clear operational way to define difficulty of any type of task. The issue is well exemplified in the review by Grassmann et al. (2016), where studies aiming to understand the cognition-respiration association have employed a mixture of approaches with very different designs and models to manipulate cognitive load or task difficulty. The review shows that some authors strictly adhere to the cognitive load theory to define difficulty level of their tasks. Accordingly, these types of studies may rely on attentional demands in one perceptual modality (usually visual) in which the number of manageable items in working memory capacity and/or number of distractors are adjusted to increase/decrease difficulty level (e.g., Forster and Lavie, 2008). Other type of studies also belonging to the load theory, rely on cognitive control, such as during car driving (e.g., Engstrom et al., 2017). In this approach, difficulty level is determined by the type and number of perceptual and mental functions needed. In spite that the referred studies are convenient for laboratory conditions and quantitatively adjust for difficulty level, the theory and its approaches have been criticized. Among the most important criticisms are the lack of a representative design, its ambiguity to manipulate load (maintenance-memory tasks vs. cognitive control tasks), and unclearness to define the concept of “load” (perceptual load vs. cognitive load) (Murphy et al., 2016).

This state of affairs demonstrates how complicated it is to define difficulty of a test and the matter turns even more complex regarding oral verbal tasks that can be used to measure respiration in real-time. Appropriate test choices for these purposes are not abundant and the existent ones are too diverse (e.g., free speech, recitation of verses, counting backwards, see Scholkmann et al., 2013). Some researchers have reflected into the issue of difficulty in speaking tasks and proposed core elements to define difficulty level (Skehan, 1998, 2009; Fulcher and Marquez Reiter, 2003). A pioneer view at this respect (Skehan, 1998) proposed that task familiarity together with variation and complexity in the mental operations involved are elements strongly determining verbal difficulty. Thus, the less familiar and the greater and varied the number of mental operations demanded, the more difficult a verbal task will be.

Based on this rationale, we propose that a type of tasks reuniting these characteristics and presenting different difficulty levels are various *naming* tests. These tests rely on recognizable visual stimuli such as letters, words, text passages, numbers, or objects that entail an expected oral answer (Kirby et al., 2010). In these tasks, participants are required to “*name”* or generate words according to the visual stimuli as quickly as possible. A rich literature about the mental processes engaged in naming tests, allows us to delineate the mechanisms involved in particular naming executions. Thus, taking into account that type and complexity of cognitive processes are determinants of verbal tasks difficulty, we decided to employ five naming tasks that share basic processes but that differ in additional mental operations, which entails a variation of difficulty between them.

*Selected naming tasks and difficulty level:* The chosen tasks already employed in our laboratory (Rodríguez-Aranda and Jakobsen, 2011) are reading of single words, reading a text passage, object naming, semantic, and phonemic verbal fluencies. The rationale to define degree of difficulty relies on two main features: automaticity and involvement of executive functions. We will first develop the automaticity aspect of the selected tasks. In line with a broad literature on naming and reading, automaticity refers to the easiness and swiftness to produce a response (Lam et al., 2017). The most automatic a task is, the faster and accurate it is performed (Wolf et al., 2000). For instance, oral production of alphanumeric stimuli (i.e., letters and numbers) is performed with lower-level demands of attentional resources and rapid responses (Wolf, 1991). In contrast, non-graphological naming, such as naming colors, objects or pictures rely on more advanced levels of processing to identify visual features and integrate perceptual and phonological mechanisms. For this reason, the responses in non-graphological naming are more demanding and occur after longer latencies (Wolf et al., 2000).

Accordingly, in our study reading of single words is the most familiar and automatized action and therefore, the easiest task. It is based on an orthographic process to recognize written characters and articulation of the word is easy for literate persons (Kirby et al., 2010). The next task that follows in difficulty is reading of a text, which requires higher cognitive and respiratory resources than single word reading as larger number of utterances and higher constraints on syntax exist (Winkworth et al., 1994). A skillful performance for reading text passages entitles vocabulary familiarity and comprehension of the text (Kirby et al., 2010). The next task following in terms of difficulty is object naming. Here, not only recognition of the stimuli and object representation are demanded but also the search of meaning, which prolongs processing time (Glaser, 1992; Wolf et al., 2000). Besides, in object naming semantic search as well as familiarity to the stimuli play a role for degree of difficulty (Bonin et al., 2002). Finally, the most demanding tasks are those designated as generative naming tests (Kozora and Cullum, 1995), also known as verbal fluency tasks (VFTs). In VFTs subjects are confronted with either a category (e.g., animals) or a letter of the alphabet (e.g., F, A, S) and they are required to produce as many words, as fast as possible, matching the category or initial-letter presented in a restricted time. The VFTs most commonly employed are the semantic and phonemic tests, which rely on semantic memory, lexical retrieval, fast processing speed, and executive functioning (Rodríguez-Aranda and Jakobsen, 2011). Degree of difficulty between VFTs is usually based on a discrepancy score where number of generated words for semantic fluency is greater than in the phonemic variant in healthy individuals (Vaughan et al., 2016). One reason for this discrepancy is that searching words based on a category (or semantic relationships) is a familiar action occurring in daily life, which is more automatized than finding words according to an initial-letter (Rosser and Hodges, 1994).

However, differences in degree of difficulty between VFTs need also to be understood in terms of their demands on executive functions (EF). This brings us to the issue of ranking difficulty level of the naming tasks based on their involvement with EF. To begin with, it is important to stress that language and EF are complex behaviors that are intrinsically related to each other and degree of relatedness varies depending on type of language action (Shokrkon and Nicoladis, 2022). As a matter of fact, automatized tasks such as reading of individual words has been reported to correlate with working memory and shifting but not with inhibitory control (Altani et al., 2017). In contrast, appropriate reading of text passages has been positively associated with overall EF abilities (Kieffer and Christodoulou, 2020). As for object naming, numerous reports point to strong correlations between single presentation of pictures and prototypical aspects of EF (i.e., working memory, shifting, and inhibition). The link between object naming and EF has been proved through composite scores of EF (Altani et al., 2017) and with single neuropsychological EF measures (Higby et al., 2019). Now, regarding VFTs, we have already stated that to different degrees, both of them require EF, as these tasks demand monitoring of generated words and inhibitory mechanisms to avoid repetitions and produce new words. However, the semantic VFT is the least engaged with EF because the central cognitive process required is the search of meaningful responses based on semantic associations (Tupak et al., 2012). The need of EF in semantic VFT is mainly related to shifting subcategories and avoidance of repeated words. In contrast, phonemic VFT is regarded as a prototype EF task due to its higher requirements on inhibitory control and search of strategies. Besides the fact that word retrieval based on an initial-letter in itself represents a demanding action, further constraints increase the involvement of EF, such as instructions to avoid generation of proper nouns, inflections, and suffixes of lexemes as well as perseverative answers (Birn et al., 2010). Consequently, the demands on inhibitory processes, searching unusual strategies and monitoring, to successfully perform phonemic VFT are greater than for semantic VFT.

Thus, collectively and based on the automatization aspect and involvement of EF in our study, the least demanding task is reading of single words, followed by reading of a text passage, object naming, semantic VFT, and phonemic VFT.

## 3. Evaluation of respiratory function and hypotheses

In the present study, we aim to explore the effects of naming tasks on respiratory parameters during the entire breathing cycle, that is, during both the inspiratory and expiratory phases. The reason is that currently, it is still unclear how the breathing phases relate to cognition and brain functioning. Even though there are some data relating each part of the breathing cycle with cognitive and cerebral activation, the findings are still limited. A search in the literature revealed some studies linking cognition and brain functioning with the inspiratory phase. It seems that deeper inspirations are required before generation of meaningful phrases and grammatically demanded expressions in spontaneous speech (Winkworth et al., 1995; Wang et al., 2010). In addition, the importance of the inspiratory phase for brain functioning has been reported in both rodents and humans (Hsu et al., 2020; Juventin et al., 2022). Supporting these findings is the study by Zelano et al. (2016) in which participants showed better memory retrieval for stimuli presented during inspirations. Although, the majority of the existent studies link inspiration with cognitive functioning, there are also data associating the expiratory phase with cognitive operations. Such is the case of the studies conducted by Waselius et al. (2019, 2022) in which associative learning through eyeblink conditioning turns out to be optimal when taking place during expiration.

The above findings show that cognitive demands may affect both phases of the breathing cycle. However, less is known about how specific airflow parameters relate to different verbal tasks. Some earlier studies have reported that volume, duration and depth of airflow are upregulated during inspiration in spontaneous speech (Winkworth et al., 1994, 1995). The authors speculated that such increments in airflow during inspiration were associated with the neural planning necessary for speaking. Apart from these data to our knowledge there are no other accounts informing about the relationship of individual airflow measurements and specific speaking tasks. Therefore, it is reasonable to expect effects of the naming tests on the inspiratory phase across all airflow parameters. If it is the case that airflow measures are upregulated by cognitive requirements, we would expect specific increments of airflow in the most demanding naming tasks and variation of airflow measures should occur proportionally to task difficulty. In addition, even though there are no data connecting airflow during expiration and cognitive functions, we wish to draw attention to a particular measure in the expiratory phase: peak expiratory airflow (PEF). This measurement has been associated in different disciplines with cognitive abilities in various populations such as: healthy older adults (e.g., Cook et al., 1995; Trevisan et al., 2020), demented patients (Paixao et al., 2021), healthy younger adults (Lehrer et al., 2003), and minorities (Allaire et al., 2007). Also, PEF is a well-known respiratory parameter frequently used to calculate an index for cardiorespiratory fitness, which is an indicator of physical and mental health. The PEF, as a marker for health status, has been used to evaluate and monitor the optimal capacities of youths (Chang et al., 2020) and high-performing athletes (Galanis et al., 2006) among others. Finally, the PEF showed to be an appropriate pointer of brain dynamics in animal research (Juventin et al., 2022). Hence, PEF is an important respiratory parameter for cognitive performance and thus, we expect that PEF in particular will appear as a significant outcome associated with the most demanding verbal tasks selected for the present investigation.

## 4. Materials and methods

### 4.1. Participants

Thirty healthy young adults (16 women, 14 men; age: M = 25.37 years, SD = 3.21) participated in the current study. Participants were recruited *via* flyers at the University of Tromsø and social media, as well as word of mouth. Inclusion criteria were being right-handed (self-reported), native Norwegian speaker, no history of head trauma, and not depressed. Participants were screened for signs of depression with Beck’s Depression Inventory II (BDI-II; Beck et al., 1996). Demographic variables were collected *via* background interview. Five participants were excluded from the study. Two of these, scored higher than the cut-off on the depression questionnaire, two reported respiratory ailments during the background interview, and one had missing data on some of the respiratory measurements. All participants were aware that participation in the study was voluntary and each of them provided signed, informed consent prior to the testing and interview session. The study was approved by the local Regional Committee for Medical and Health Research Ethics (REK).

### 4.2. Materials

Interview. Each session included a semi-structured interview to collect information regarding background information such as, health issues (e.g., use of medications, exercise, and smoking habits) and demographic variables. General subjective health was rated by asking participants to range their own health as either bad, medium, or good.

Beck’s Depression Inventory II (BDI-II) (Beck et al., 1996). BDI-II was used to screen participants for signs of depression. The standard cut off >13 was applied.

**Figure 1:**
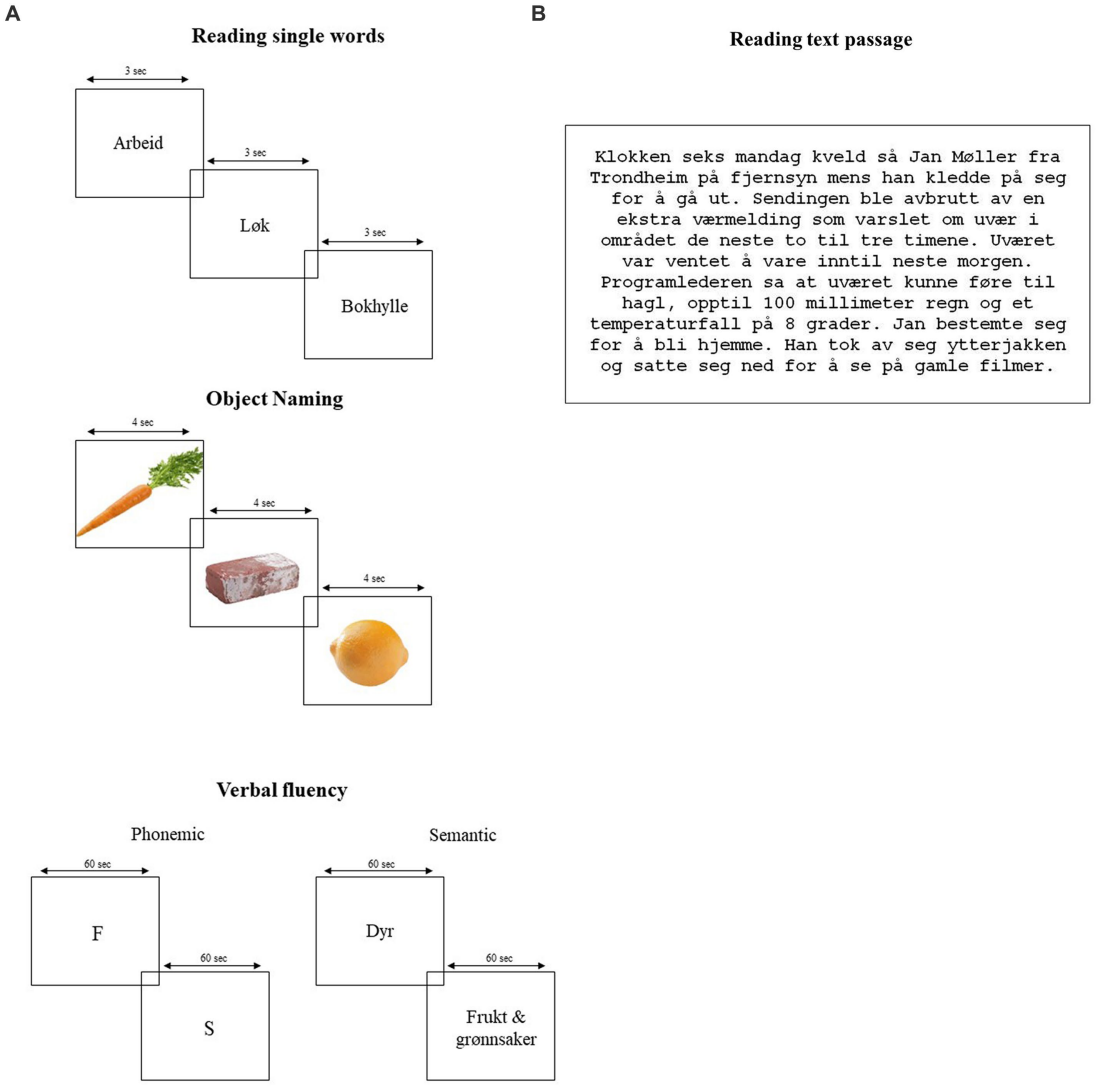
**(A)** Illustration of reading single words (top), object naming (middle), and phonemic (left bottom) and semantic (right bottom) verbal fluency. Length of stimuli presentation is displayed on the top of each word/figure/letter/category. **(B)** Illustration of the reading text passage task in Norwegian. The text was displayed on screen for 60 s.

*Verbal tasks.* Five naming tasks were employed and adapted to be presented on a 19” Dell monitor with the software E-prime 2.0 (Psychology Software Tools, Inc., Pittsburgh, PA, USA). This set of tasks is the same utilized as in our early study (Rodríguez-Aranda and Jakobsen, 2011). The only addition that we deemed important to make on the present study was the implementation of reading of a text passage as this task is not cognitively demanding but entitles connected speech, which has been addressed in previous investigations on speech breathing. Also, the design for verbal tasks was the same as the one used in 2011, in which duration for stimuli presentation of each verbal task was determined upon pilot trials and stimuli presentation was controlled by the experimenter. The exogenous control of stimuli presentation is a convenient solution as participants only need to look at the monitor and generate their answers. In this way, additional constraints related to voluntary actions are avoided, especially since participants need to maintain the pneumotachograph mask in place by the use of both hands. An illustration of the employed tasks is presented in Figures 1A,B.

*Reading of single words.* A simple reading task consisted of 10 regular and frequent Norwegian words of various lengths presented in a 45-point Courier New font, every 3,000 ms. in the center of the screen. Participants were instructed to read the words aloud as fast as they appeared on the screen (see Figure 1A).

*Reading text passage.* A short story from the Logical memory I, from the Wechsler Memory Scale III (WMS-III, (Wechsler, 1997), was displayed in full on the computer screen for 60 s. The short story consisted of 6 sentences and a total of 87 words. Participants were instructed again to read aloud as quickly as possible the whole text and try not to commit errors (see Figure 1B).

*Object naming*. This task consisting of 15 pictures of animals and objects from the Naming Test of the Neuropsychological Assessment Battery (NAB) were presented, one by one, in the center of the computer screen (Stern and White, 2003). In this task, the pictures were presented at a fixed interval of 4,000 ms. Participants were instructed to name aloud the pictures as fast as possible in the span of 60 s (see Figure 1A).

*Verbal fluency tasks.* Two tasks were used to assess verbal fluency: Phonemic and semantic fluencies. The phonemic task was an adaptation of the Controlled Oral Word Association Test (COWAT) (Benton, 1967). Participants were instructed to generate as many words as possible for 60 s starting with a specific letter of the alphabet presented on the screen. The letters “F” and “S” were selected. Specific rules for this task were: avoid proper names, variants of a word (e.g., book, bookshelf), and repetitions. The semantic fluency task included two different categories, namely “animals” and “fruits & vegetables.” As with the phonemic task, the semantic task also lasted 60 s and again participants were instructed to produce as many words as possible during the given time. This time, the only rule was to avoid repetitions (see Figure 1A).

#### 4.2.1. Instrumentation, measurements, and protocol for assessment of respiratory function in the verbal tasks

*Apparatus:* The Kay Elemetrics Phonatory Aerodynamic System (PAS) (model 6600, KayPENTAX Elemetrics, Lincoln Park, NJ) was used. The PAS consists of a circumferentially vented pneumotachograph mask with integrated microphone placed at 15 cm distance from the mouth, which allows simultaneous acquisition of acoustic and aerodynamic data. Thus, we registered three aerodynamic parameters during expiratory and inspiratory periods: Airflow duration, peak airflow, and airflow volume. These measurements were taken during vital capacity protocols (see below) and during execution of the verbal tasks. Acquisition of vital capacity data was deemed necessary in order to corroborate appropriate lung capacity of our participants. Regarding acoustic data, all oral responses emitted during the execution of verbal tasks were recorded and used in subsequent analyses for scrutiny of the content.

*Vital Capacity (VC) protocol.* The maximum volume of air expired after a maximum inspiration (Miller et al., 2005) was measured. We employed a standard protocol for forced vital capacity, which was recorded with the PAS. Before each session, the spirometer was calibrated with a 1 L syringe to ensure accuracy of the respiratory measurements. Participants were instructed to sit upright with both feet touching the floor to get the best performance possible. The PAS device was held comfortably by the participants themselves completely covering nose and mouth with the mask, making sure there was no air leakage during the procedure. Instructions were to inhale maximum, position the mask tightly on the face by holding the breath for a second, and then exhaling as forcefully as possible until they felt they could not exhale any more. The experimenter assured that all participants clearly understood the instructions before data acquisition started. A minimum of three vital capacity maneuvers were carried out. Adequate pauses between each trial were given.

**Figure 2:**
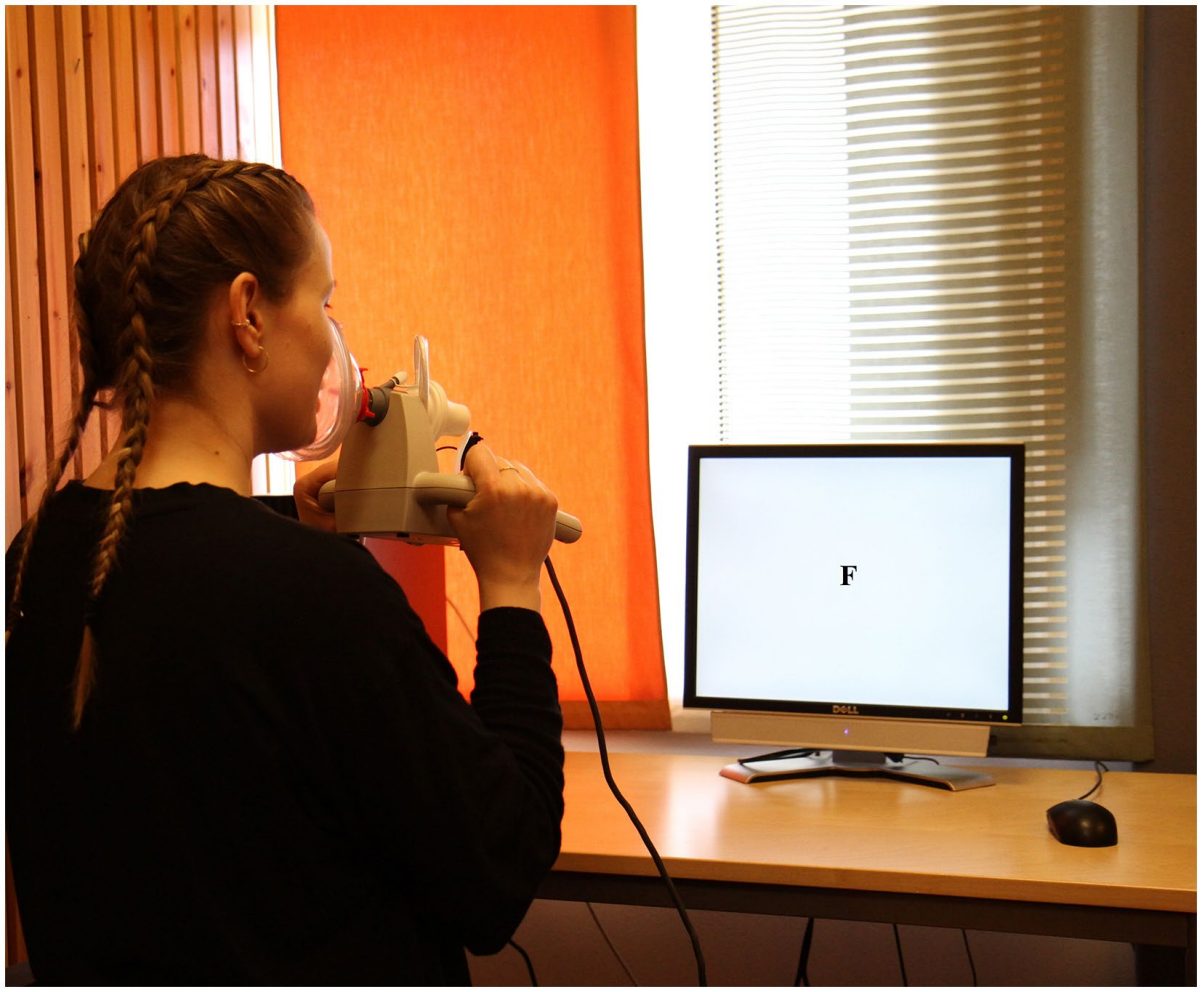
Display of the experimental setup.

*Protocol for acquisition of respiratory function during verbal tasks.* The five selected verbal tasks were assessed after the last vital capacity maneuver. Participants were faced toward the computer screen (See Figure 2). As in the vital capacity procedure, participants held the PAS device comfortably against their faces, covering both mouth and nose before starting to execute the tasks. Duration of all verbal tasks was of a maximum of 1 min and instruction for all of them was to perform the given task as fast as possible. However, the total number of produced words during a minute differed strongly across tasks, specifically for reading a text passage and for VFTs. In the former, the time needed to read the whole passage varied considerably, and most often participants finished the test in less than 30 s. As for VFTs, the highest number of words generated takes place during the first 15 s (Crowe, 1998). Thus, based on the above, we decided to analyze *in extenso* respiratory data of the first 15 s of each task. However, we also explored the stability of respiratory demands by comparing the first 15 s (i.e., 0–15 s. called thereafter “interval 1”) against the following 15 s-interval (i.e., 16–30 s, called thereafter “interval 2”). We ensured that short pauses were given between each of the verbal tests allowing for participants to rest and for the necessary auto-zeroing of the spirometer to obtain valid recordings.

#### 4.2.2. General procedure

The assessments took place at the department of Psychology at the University of Tromsø. Written and oral information about the study and procedures were provided to each participant prior to the testing session. All participants signed an informed consent, and they were free to withdraw from the testing at any point. The total duration of the testing procedure was about 1.5 h. The initial background interview was first conducted, followed by the BDI-II. Since the present study belongs to a larger project, a neuropsychological battery was also applied. However, the cognitive data were not included for the current study. Afterwards, the vital capacity protocol was executed, followed by the verbal tasks in the following fixed order: Phonemic VFT, semantic VFT, object naming, reading of single words, and reading a text passage. The fix order of the tasks was deemed necessary to avoid possible priming effects on VFTs.

#### 4.2.3. Statistical analyses

A repeated-measures MANOVA was carried out to investigate the various respiratory measures during the five different verbal tasks: reading of single words, reading text passage, object naming, semantic and phonemic VFTs. Bonferroni-corrected pairwise comparisons were conducted to follow-up significant main effects. IMB SPSS Statistics version 28 was used for statistical analyses (IBM Corp., Armonk, N.Y., USA).

## 5. Results

### 5.1. Demographics and background variables

Demographic and background data are presented in Table 1. Results showed that participants had a mean of 16.77 (SD = 2.16) years of formal schooling and their scores in the BDI questionnaire indicated that none of them were depressed. Furthermore, the sample’s vital capacity results are in line with what is expected for healthy individuals of matching mean age (Zraick et al., 2012).

**Table 1:** Demographic and background characteristics.

	Sample young adults (*n* = 30)
F/M ratio	16/14
	*M (SD)*
Age (years)	25.37 (3.21)
Education (years)	16.77 (2.16)
BDI (mean score)	3.67 (2.88)
Vital Capacity (mean values)	
Expiratory airflow duration (s)	4.18 (1.83)
Peak expiratory airflow (L/s)	9.05 (3.55)
Expiratory volume (L)	5.16 (1.27)

*n* = sample size, M, mean; SD, standard deviation; BDI, Beck’s depression inventory.

### 5.2. Accuracy in verbal tasks

Results regarding the accuracy of performance on each task during the first 15 s showed that participants produced on average 4.97 words (SD = 0.18) during reading single words; 47.17 words (SD = 6.06) during reading a text passage, 3.77 words (SD = 0.43) during object naming, 8.48 words (SD = 1.60) for semantic VFT and 5.85 words (SD = 1.17) for phonemic VFT. The average length for reading the text passage was 26.99 s with a range of 20.14–35.07 s. All participants completed the reading text passage task without any errors. As it is expected from healthy younger adults; almost no errors were committed across tasks. The few errors observed occurred in object naming (*M* = 0.17, SD = 0.38) in terms of incorrect words and in the VFTs (*M* = 0.05, SD = 0.16) in terms of repetitions or words not belonging to the category/letter presented.

### 5.3. Airflow measurements

The repeated measures MANOVA contrasting the multiple respiratory variables during the five verbal conditions (reading single words, reading text passage, object naming, semantic and phonemic verbal fluencies) showed a significant main effect of type of test (Pillai’s Trace = 1.39, *F* (24, 456) = 10.08, *p* < 0.001, η_p_^2^ = 0.35). The univariate *F* tests further confirmed the existence of significant differences across tasks on all respiratory measures (see Table 2).

**Table 2:** Means, standard deviations and univariate statistics for respiratory measures by verbal task.

	Reading single words		Reading text passage		Object Naming		Semantic Fluency		Phonemic Fluency		*F* (*4,116*)	*p value*	*η_p_^2^*
	*M*	*SD*	*M*	*SD*	*M*	*SD*	*M*	*SD*	*M*	*SD*			
Insp. Airflow duration	5.08	0.98	2.50	0.76	5.12	0.80	4.34	0.90	4.62	0.89	56.79	*p* < 0.001	0.66
Exp. Airflow duration	9.90	0.98	12.50	0.76	9.85	0.80	10.58	0.89	10.32	0.89	58.48	*p* < 0.001	0.67
Peak Insp. Airflow	−0.89	0.32	−2.18	0.81	−0.90	0.39	−1.47	0.44	−1.41	0.45	44.08	*p* < 0.001	0.60
Peak Exp. Airflow	0.65	0.23	0.69	0.28	0.67	0.28	1.00	0.50	1.06	0.58	13.57	*p* < 0.001	0.32
Insp. Volume	−1.83	0.65	−1.64	0.67	−1.78	0.57	−2.22	0.72	−2.36	0.77	9.80	*p* < 0.001	0.25
Exp. Volume	1.97	0.58	1.66	0.50	1.97	0.71	1.90	0.64	2.02	0.87	2.41	*p* = 0.053	0.08

M, mean; SD, standard deviation; Insp, inspiratory; Exp, expiratory.

#### 5.3.1. Pairwise comparisons by respiratory parameter

##### 5.3.1.1. Airflow duration

**Figure 3:**
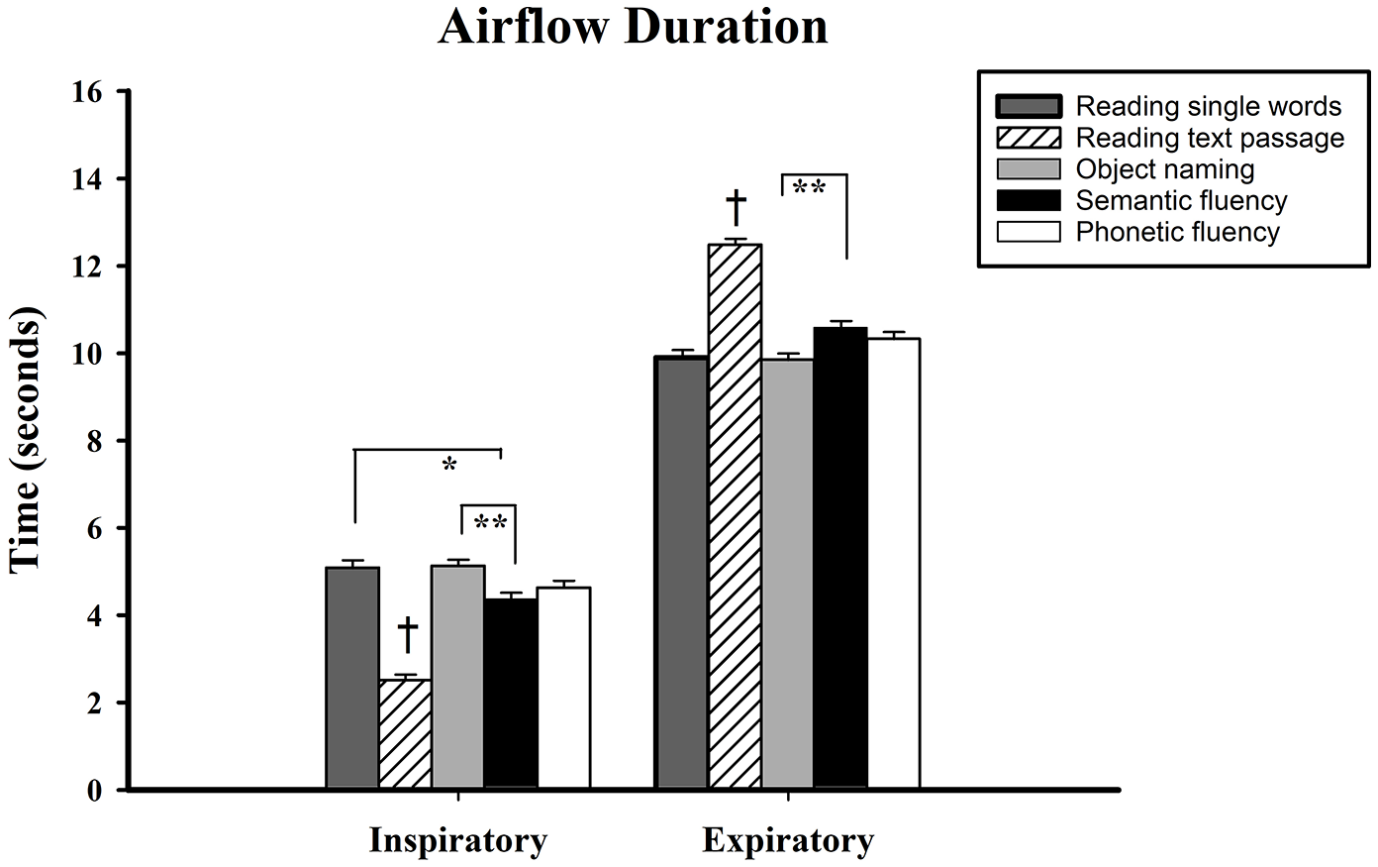
Airflow duration measured in seconds, shown on vertical axis. Verbal tasks shown on horizontal axis. † = different from all tasks; *** < 0.001, ** < 0.01, * < 0.05. Standard error displayed in error bars. Measurements were conducted on the first 15 s of each task.

Follow-up comparisons with the Bonferroni correction showed that when reading a text passage, the inspiratory airflow duration was significantly shorter in this task than in all other tests (reading single words M_diff_ = −2.58, *p* < 0.001; object naming M_diff_ = −2.62, *p* < 0.001; semantic fluency M_diff_ = −1.85, *p* < 0.001; phonemic fluency M_diff_ = −2.12, *p <* 0.001). Furthermore, a significant difference was found between object naming and semantic fluency (M_diff_ = 0.77, *p* < 0.01) in which the latter had shorter inspiratory duration. Interestingly, no difference existed between object naming and phonemic fluency. Regarding expiratory airflow duration, pairwise analyses showed that reading a text passage entailed the longest expiratory time as compared to all other tasks (reading single words M_diff_ = 2.57, *p* < 0.001; object naming M_diff_ = 2.63, *p* < 0.001; semantic fluency M_diff_ = 1.91, *p* < 0.001; phonemic fluency M_diff_ = 2.16, *p <* 0.001). Additionally, semantic fluency required significantly larger expiratory duration than the object naming task (M_diff_ = 0.73, *p* < 0.01) (see Figure 3).

##### 5.3.1.2. Peak airflow

**Figure 4:**
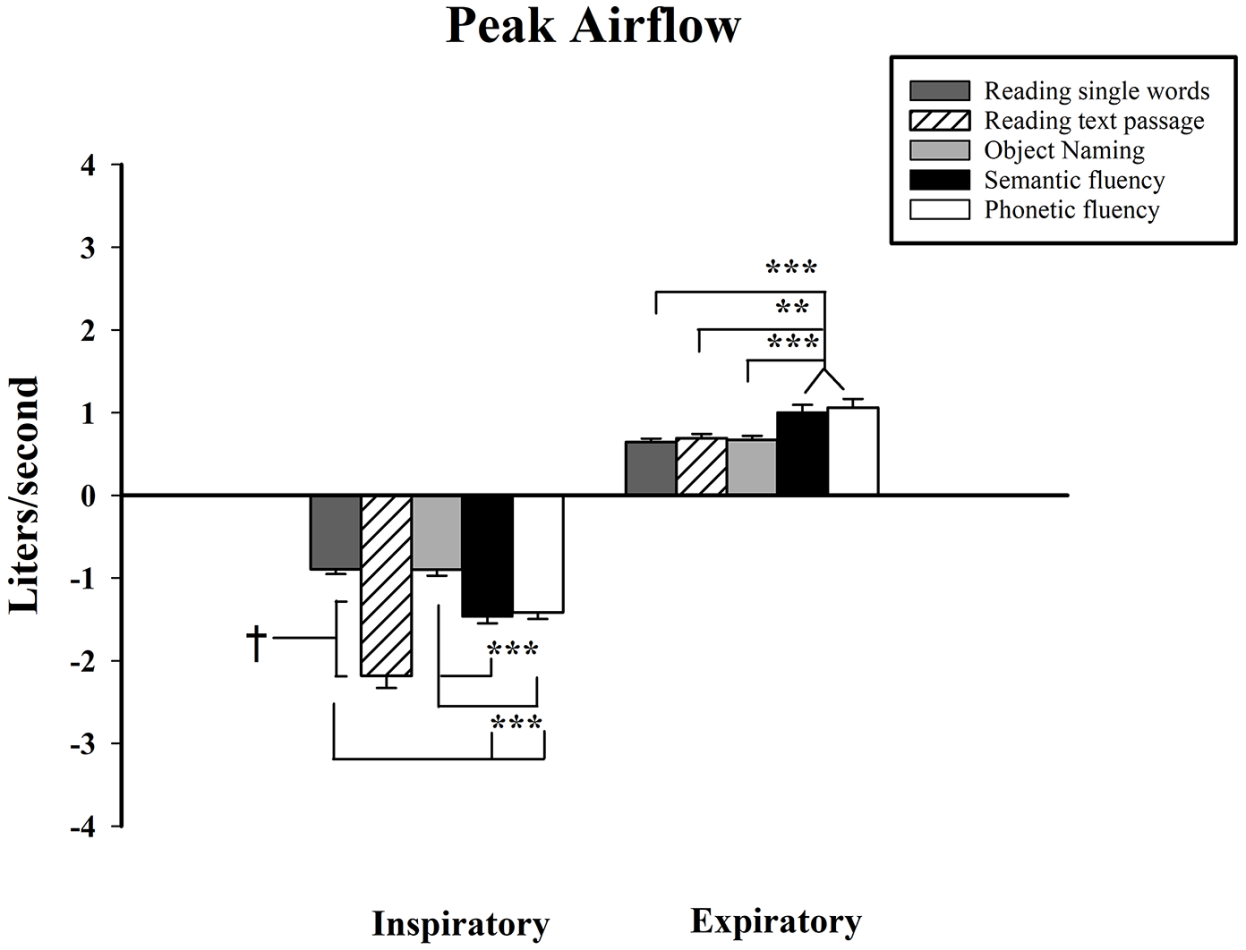
Peak airflow measured in liters per second, shown on vertical axis. Verbal tasks shown on horizontal axis. *** < 0.001. Standard error displayed in error bars. Measurements were conducted on the first 15 s of each task.

Pairwise comparisons showed that during the inspiratory phase, the peak airflow was significantly larger during reading a text passage as compared to the rest of the verbal tests (reading single words M_diff_ = −1.29, *p* < 0.001; object naming M_diff_ = −1.28, *p* < 0.001; semantic VFT M_diff_ = −0.72, *p* < 0.001; phonemic VFT M_diff_ = −0.77, *p* < 0.001). Moreover, significant differences were found between both fluency tasks and reading single words (semantic M_diff_ = −0.57, *p* < 0.001; phonemic M_diff_ = −0.52, *p* < 0.001) and object naming (semantic M_diff_ = −0.57, *p* < 0.001; phonemic M_diff_ = −0.51, *p* < 0.001) also during inspiration in which the peak value was larger for both fluency tasks. Thus, results showed that a greater peak of inspiratory airflow, i.e., more liters inhaled per second, existed for the reading text passage task and secondly for VFTs. As for the expiratory phase, peak airflow was only significantly different between both VFTs and the rest of the tasks. For the semantic VFT the following significant differences were found against reading single words M_diff_ = 0.36, *p* < 0.001; reading a text passage M_diff_ = 0.31, *p* < 0.01; object naming M_diff_ = 0.33, *p* < 0.01. Likewise, the phonemic VFT showed significant differences against reading single words M_diff_ = 0.41, *p* < 0.001; reading a text passage M_diff_ = 0.36, *p* < 0.01 and object naming M_diff_ = 0.39, *p* < 0.001. Thus, both VFTs displayed similar expiratory peak values and were notoriously higher than the other verbal tests (see Figure 4).

##### 5.3.1.3. Airflow volume

**Figure 5:**
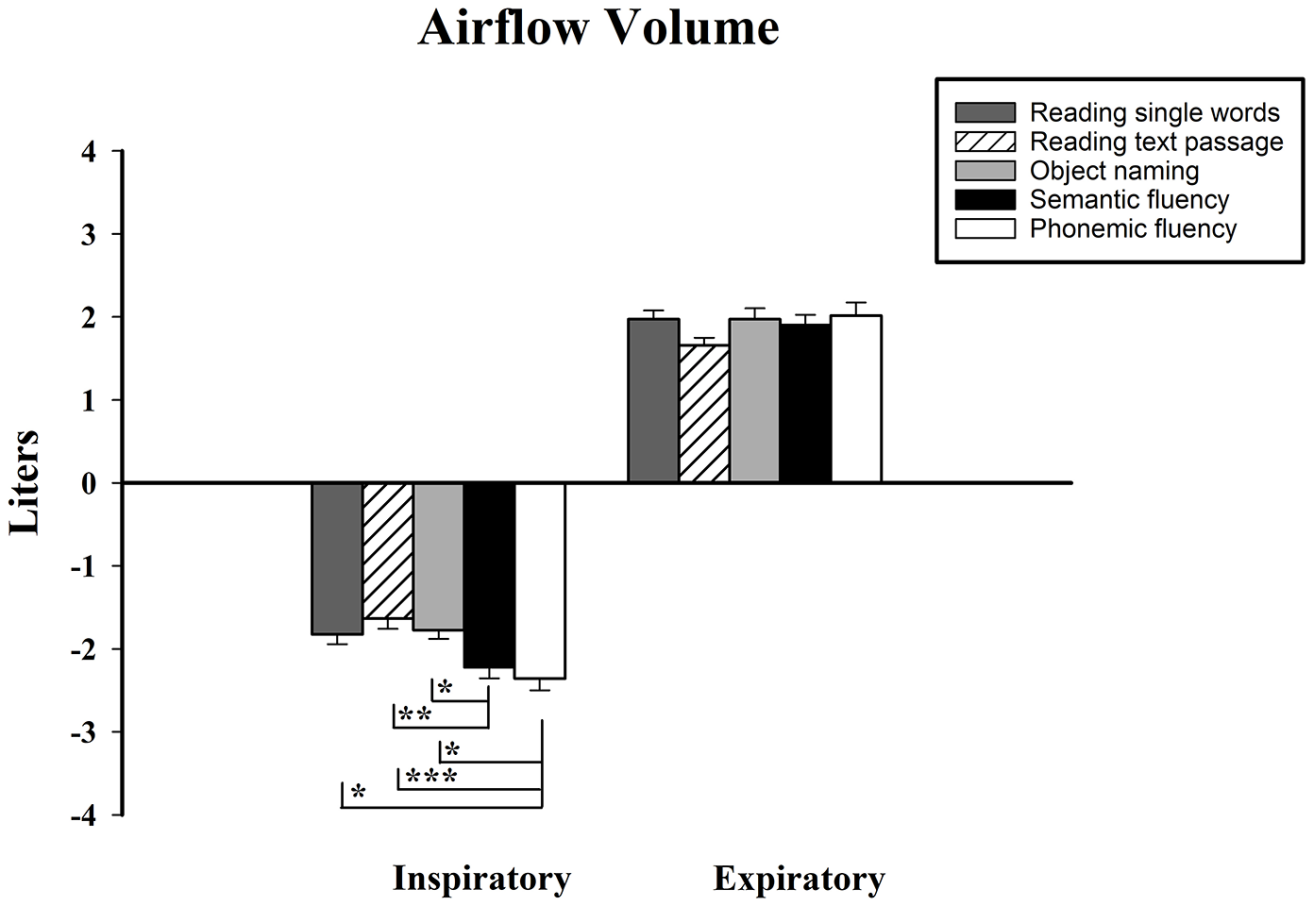
Volume measured in liters, shown on vertical axis. Verbal tasks shown on horizontal axis. † = different from all tasks; *** < 0.001, ** < 0.01. Standard error displayed in error bars. Measurements were conducted on the first 15 s of each task.

In this respiratory measurement (see Figure 5), pairwise statistics showed that during the inspiratory phase, significant differences were present between the VFTs and the other tests. The inspiratory volume for semantic VFT was significantly greater than during reading a text passage task (M_diff_ = −0.59, *p* < 0.01), as well as in the object naming task (M_diff_ = −0.45, *p* < 0.05). As for phonemic VFT, differences in the inspiratory volume were found against reading single words (M_diff_ = −0.53, *p* < 0.05), reading a text passage task (M_diff_ = −0.72, *p* < 0.001), and the object naming task (M_diff_ = −0.58, *p* < 0.05). Thus, both VFTs required a greater volume of inspiration than the other verbal tasks. Concerning the results of expiratory volume, no significant differences were found across the five tasks.

#### 5.3.2. Test comparisons adjusting number of words in reading text passage

Due to the nature of reading a text passage for 15 s, the total number of generated words was greater than in the other tasks. For this reason, we decided to conduct a new repeated measures MANOVA with comparable number of words across tasks. Hence, we contrasted once more, respiratory outcomes when 6 and 10 words were generated during reading in connected speech. Results for both alternatives were almost identical and thus, only data for 10 words are presented in Supplementary Table S1. These results demonstrated again a significant main effect of type of verbal task (Pillai’s Trace = 1.65, *F* (24, 456) = 13.36, *p* < 0.001, η_p_^2^ = 0.41). Pairwise comparisons showed that for reading a text passage there was a significantly shorter airflow duration in both inspiratory and expiratory phases than in the other tasks (inspiratory: reading single words M_diff_ = −4.13, *p* < 0.001; object naming M_diff_ = −4.17, *p* < 0.001; semantic VFT M_diff_ = −3.40, *p* < 0.001; phonemic VFT M_diff_ = −3.67, *p* < 0.001; expiratory: reading single words M_diff_ = −6.27, *p* < 0.001; object naming M_diff_ = −6.22, *p* < 0.001; semantic VFT M_diff_ = −6.95, *p* < 0.001; phonemic VFT M_diff_ = −6.70, *p* < 0.001, see Supplementary Figure SA). Interestingly, results for peak airflow with 10 words during reading a text passage showed very similar findings than those from the initial analysis. In, fact there were significant differences during the inspiration phase between reading a text passage and reading single words (M_diff_ = −0.39, *p* < 0.001) and between reading text passage and object naming (M_diff_ = −0.39, *p* < 0.001). Also, significant differences were found between both VFTs and reading single words (semantic M_diff_ = −0.57, *p* < 0.001; phonemic M_diff_ = −0.52, *p* < 0.001) and both VFTs and object naming (semantic M_diff_ = −0.57, *p* < 0.001; phonemic M_diff_ = −0.51, *p* < 0.001). Results of peak airflow during expiration were the same as those encountered in the initial analyses (see Supplementary Figure SB). Finally, results for inspiratory airflow volume during reading a text passage changed drastically as significant differences were observed not only between this test and both VFTs (semantic M_diff_ = 1.66, *p* < 0.001; phonemic M_diff_ = 1.79, *p* < 0.001), but also between reading a text passage and reading single words (M_diff_ = 1.26, *p* < 0.001) and object naming (M_diff_ = 1.21, *p* < 0.001). The same was true for the expiratory phase in which 10 words during reading a text passage required significantly less airflow volume than for the other tasks (reading single words M_diff_ = −1.38, *p* < 0.001; object naming M_diff_ = −1.39, *p* < 0.001; semantic VFT M_diff_ = −1.32, *p* < 0.001; phonemic VFT M_diff_ = −1.43, *p* < 0.001; see Supplementary Figure SC).

#### 5.3.3. Stability of respiratory demands through time and representativeness of time window

To understand whether the respiratory demands varied over time, we divided the first 30 s of test performance of all tasks into two 15 s-intervals (0–15 s. = interval 1, and 16–30 s. = interval 2) and we conducted paired t-test to compare airflow outcomes between intervals 1 and 2.

We remind the reader that enough data existed for all tests during the first 30 s, which enabled the assessment of respiratory stability during word production. This yields the reading of text passage in which subjects finished to read the text in less than 30 s., and even in both VFTs where the mean of generated words during the first two intervals were the most productive (interval 1: semantic = 8.48 (SD = 1.6), phonemic = 5.85 (SD = 1.7); interval 2: semantic = 5.67 (SD = 1.59), phonemic = 3.78 (SD = 1.45)) as compared to the last 15-s. intervals (interval 3: semantic = 4.03 (SD = 2.06), phonemic = 3.43 (SD = 1.12); interval 4: semantic = 3.08 (SD = 1.14), phonemic = 2.5 (SD = 1.13)).

Thus, results of the comparison between intervals 1 and 2 showed a large variability across tasks between both intervals (see Table 3). It appeared that airflow volume was the most unstable of all respiratory parameters as all the tasks showed at least one significant difference on this outcome. Significant changes in airflow volumes occurred during the two reading tasks at both phases of the respiratory cycle (all at *p < 0*.001, except for expiratory volume for reading single words, *p <* 0.01). Similarly, changes in volume were observed during inspiration for object naming (*p < *0.001) and phonemic VFT (*p < *0.05), while the semantic VFT showed the equivalent change in the expiratory phase (*p < *0.01). Regarding airflow duration, significant changes between interval 1 and 2 occurred for reading a text passage and object naming across the two phases of the respiratory cycle (*p < *0.001). Finally, peak airflow showed changes only during inspiration.

**Table 3:** Paired-sample t-tests comparing airflow outcomes between interval 1 and 2.

Variable	Interval 1 (0–15 s)	Interval 2 (16–30 s)	*t*	*p* (two-tailed)
Insp. Airflow Duration				
Reading words	5.10 (0.97)	5.30 (0.68)	−1.32	NS
Reading text	2.50 (0.76)	1.60 (0.60)	6.84	<0.001
Object Naming	5.10 (0.80)	5.80 (0.77)	−4.24	<0.001
Semantic VFT	4.40 (0.90)	4.20 (0.91)	−0.80	NS
Phonemic VFT	4.60 (0.89)	4.50 (0.77)	−0.85	NS
Exp. Airflow Duration				
Reading words	9.90 (0.97)	9.70 (0.69)	1.19	NS
Reading text	12.50 (0.76)	9.70 (2.70)	4.78	<0.001
Object Naming	9.80 (0.80)	9.20 (0.77)	4.25	<0.001
Semantic VFT	10.60 (0.88)	10.70 (0.88)	−0.91	NS
Phonemic VFT	10.30 (0.89)	10.50 (0.77)	−0.87	NS
Peak Insp. Airflow				
Reading words	−0.89 (0.31)	−0.96 (0.36)	1.32	NS
Reading text	−2.20 (0.80)	−1.90 (0.70)	−1.30	NS
Object Naming	−0.90 (0.39)	−0.83 (0.45)	−0.62	NS
Semantic VFT	−1.50 (0.44)	−1.30 (0.48)	−2.12	<0.05
Phonemic VFT	−1.40 (0.44)	−1.20 (0.49)	−3.99	<0.001
Peak Exp. Airflow				
Reading words	0.64 (0.23)	0.73 (0.28)	−1.99	NS
Reading text	0.69 (0.28)	0.76 (0.36)	−1.55	NS
Object Naming	0.67 (0.28)	0.69 (0.36)	−0.46	NS
Semantic VFT	1.00 (0.50)	1.10 (0.48)	−1.17	NS
Phonemic VFT	1.10 (0.57)	1.10 (0.68)	−0.08	NS
Insp. Volume				
Reading words	−1.80 (0.65)	−2.25 (0.61)	5.67	<0.001
Reading text	−1.63 (0.67)	−1.10 (0.58)	−3.63	<0.01
Object Naming	−1.80 (0.57)	−2.21 (0.69)	4.21	<0.001
Semantic VFT	−2.22 (0.71)	−2.10 (0.69)	−1.11	NS
Phonemic VFT	−2.40 (0.77)	−2.10 (0.78)	−2.63	<0.05
Exp. Volume				
Reading words	1.97 (0.58)	2.22 (0.70)	−3.11	<0.01
Reading text	1.66 (0.50)	1.32 (0.59)	3.51	<0.01
Object Naming	1.97 (0.71)	2.00 (0.68)	−0.26	NS
Semantic VFT	1.91 (0.64)	2.22 (0.79)	−3.63	<0.01
Phonemic VFT	2.01 (0.86)	2.20 (0.88)	−1.98	NS

Mean (standard deviations).

In sum, analyses on the stability in respiratory demands by task showed that reading a text passage and object naming were the most unstable tests. Thereafter, VFTs showed changes in peak airflow and volume, while reading of single words showed changes only in airflow volume.

To round off the issue of stability, it seems prudent to look at the effects of the selected time window. As explained in the protocol of acquisition of respiratory measures, we decided that a 15 s interval was an appropriate period, due to the more abundant word generation in VFTs in this interval. However, it is possible that a larger time window would show important differences regarding the representativeness of our data. In order to assess representativeness of the results, we obtained the respiratory values for the first two intervals altogether, that is for the whole first 30 s. The results are presented in Supplementary Table S2. These data demonstrated that to some extent, time window matters, mainly regarding the variables of duration and volume where values practically doubled in most of the tasks as compared to the initial data from 15 s. These results are reasonable as the time is longer and data is proportionally cumulated. However, the 30-s. analysis showed that data for some of the tasks changed. For example, reading a text passage presented important differences from 15 to 30 s. on expiratory airflow duration, which was reduced when analyzing all 30 s. This change was most probably due to the fact that reading of the text was accomplished in less than 30 s and thus, we did not have the same amount of data in the two 15 s. intervals. Another difference related to expiratory duration was found for VFTs where the values were somewhat higher in the 30-s. analysis, which we also believed was related to different amount of data. These changes entailed new significant contrasts between tests, especially regarding both VFTs versus reading of single words (semantic M_diff_ = 1.77, *p* < 0.001; phonemic M_diff_ = 1.26, *p* < 0.01) and object naming (semantic M_diff_ = 2.63, *p* < 0.001; phonemic M_diff_ = 2.12, *p* < 0.001). As for the results of the inspiratory phase in airflow duration, they also demonstrated higher values but significant test differences were the same as in the original results from analysis of 15 s.

In line with the duration data, the airflow volumes were doubled for reading of single words and object naming, while this was not the case for reading a text passage and the VFTs. Thus, the duration and volume data showed that results were representative basically for the time-paced tasks. Interestingly, the results regarding peak of airflow for the 30 s. were only slightly changed, preserving all significant test contrasts as in the 15 s. analysis.

All in all, the above findings showed that reading of single words and object naming were the tests having the best representativeness in terms of time windows, while VFTs and reading a text passage, did not show equivalent results for airflow duration and volume in the 15 and 30 s. periods. Notwithstanding, peak airflow data turned out to be rather stable and representative for VFTs and reading a text passage.

## 6. Discussion

In the present study we investigated whether five verbal tasks with varying degree of cognitive difficulty entailed different respiratory airflow requirements. Overall, data showed that breathing requirements were in fact different across tasks. The most distinctive airflow outcomes were observed for reading of a text passage and verbal fluency tasks. In contrast, airflow results for reading of single words and object naming were very similar.

The fact that reading a text passage engaged so distinctive breathing needs is not surprising, as word generation in 15 s entails effortless and rapid production of words. In fact, the mean number of words produced during 15 s in this task was of 47.17, which contrasted with 4 to 7 words produced during the same period of time in the other tasks. Thus, due to the high number of utterances produced and the lack of errors registered during reading a text passage, respiratory outcomes showed extreme airflow values and notable respiratory patterns, especially during inspiration where we observed the shortest airflow duration and the largest peak airflow. Also, this task entailed the longest airflow duration during expiration. All these results agree with early research from healthy young participants reading text passages of different lengths (Lewandowski et al., 2018). These reports have demonstrated that connected speech during reading is a demanding task in terms of the physical effort exerted by the respiratory system and that breathing adjustments occur depending on the length and syntactic demands of the text (Winkworth et al., 1994). Clearly, our ancillary analyses in Supplementary material based on airflow outcomes for 6 and 10 words during reading the text passage confirm this assertion.

However, reading aloud continuously is not entirely a mechanical action. Despite that connected speech during reading exerts high demands on respiratory resources, it also relies on complex neural operations coordinating breathing and motor control during vocalization (Simonyan, 2014). Past research suggests that reading aloud engages a series of preparative actions necessary to generate timely and accurate utterances (Winkworth et al., 1994). Including in these operations are, the pre-scanning of the text that enables planning of respiratory maneuvers and the decision on the amount of air to be inhaled (Winkworth et al., 1995). Thus, our data corroborate that depending on the length of the text, there are adjustments in breathing that take place almost automatically, since reading aloud is a well-practiced action in literate younger adults.

Data from reading a text passage illustrates the interplay between breathing and cognitive control in connected speech in a relatively low demanding task. Inclusion of this task in the present study was deemed important since most research on speech breathing has been conducted on similar tasks of connected speech. However, our study also examined airflow outcomes of four additional tasks (reading single words, object naming, semantic and phonemic VFTs) in which generation of single words was required. Of these tasks, the VFTs are particularly noticeable as they were considered the most difficult tasks and entailed unique airflow requirements. Above all, the VFTs showed the highest peak airflow during expiration of all tasks, even compared to reading a text passage. Besides, in the inspiratory phase, VFT had the highest airflow volume across all four tasks and a significant high peak airflow when contrasted against reading single words and object naming.

These findings suggest that the high cognitive requirements to perform VFTs demands greater lung volume inspired, together with high peak airflows at both phases of the respiratory cycle. Data from a previous investigation evaluating breathing patterns during reading a text passage may help understand the findings on VFTs (Winkworth et al., 1994). In that study, healthy subjects adjusted their inhaled air volume in accordance with utterance length and linguistic complexity of the text. By doing so, participants displayed higher air volumes just before the appearance of semantic structures, which suggests that more air is needed at higher linguistic demands. On the same line of investigation, another study evaluating respiratory function of healthy young women during spontaneous speech, demonstrated a significant increment on initial lung volumes when “fluent” or meaningful discourse was generated (Winkworth et al., 1995). Specifically, when subjects produced complete meaningful sentences or clause boundaries, higher air volumes and longer duration of breaths preceded the execution. Conversely, when dysfluencies occurred (i.e., repetitions, meaningless sentences, filler words) smaller inspirations and expirations were observed. Taken together, the above reports support the findings of the present study in which performance on VFT required higher levels of inhaled airflow volume coupled to high peak airflow levels. Although, word production in VFT does not equal spontaneous speech, it is evident that both types of verbal actions are comparable, as performance on VFTs relies on the free production of meaningful utterances following semantic and phonemic rules.

We regard VFT’s respiratory outcomes as the most salient finding of our study, since it shows that the verbal tasks with the highest cognitive demands entailed important amounts and depth of airflow. Because VFTs were the only tasks in which PEF was significantly higher, we can confirm our initial hypothesis on the involvement of PEF in tasks with high cognitive demands. Thus, the unique relationship in our study between VFTs and high PEF needs to be understood as a consequence of high cognitive difficulty, which agree with a large body of literature linking PEF to cognition. As mentioned in the introduction, PEF is an important measure of health and cognitive status in pathological conditions (e.g., Leving et al., 2022), as well as a good index of optimal health status and higher cognitive performance in humans and animals (Galanis et al., 2006; Byrd et al., 2020; Juventin et al., 2022). Notwithstanding, to the best of our knowledge no previous research has ever linked higher levels of PEF to a concrete cognitive task. Thus, our study is the first one demonstrating the effects of complex tasks like VFTs on PEF and also, the first one giving an account about how respiratory function supports performance of controlled verbal execution with varying degrees of difficulty. However, our data related to reading of single words and object naming were not fully conclusive. In these tasks, all airflow measurements were equal at both stages of the respiratory cycle. A visual exploration of the graphical data show that only results on expiratory duration presented higher values than during inspiration, albeit the same trend yields across all tasks. There are at least three possible explanations for the lack of differences in respiratory outcomes between these two tasks. The first one is related to the time control of stimuli presentation. It is possible that by pacing presentation of words and pictures, the effects on respiratory function cannot be noticeable. A second more likely alternative, is that for healthy younger adults reading of single words and naming pictures are of equal difficulty, and therefore, similar airflow requirements were obtained. However, we rather advocate for a third explanation as a prime cause for the inconclusive data on reading single words and object naming, which relates to the instrumentation employed. Since object naming has been previously related to optimal respiratory function (Cahana-Amitay et al., 2018) and it engages quite distinctive cognitive operations, it would be reasonable to expect respiratory parameters differing from those related to reading words. Thus, the fact that we did not observe particular respiratory patterns during object naming can be related to the use of a pneumotachograph mask. This instrument samples the airflow during speaking through the mouth and nose and some studies suggest that the route (i.e., nasal vs. oral) on which the airflow is inhaled has an impact on cognition. For instance, it has been reported that nasal breathing improves learning of conditioned stimuli (Waselius et al., 2019) and that it also enhances memory retrieval (Zelano et al., 2016). Since object naming actually relies on semantic associations and lexical retrieval (Balthazar et al., 2008) it is possible that airflow data from nasal respiration would better distinguish the respiratory requirements for object naming.

Finally, the analyses on stability of respiratory demands through time (comparisons of the first two 15-s intervals) corroborated what many researchers have already stated, that a hallmark of spoken language and breathing is their extreme variability (Winkworth et al., 1994; Vlemincx et al., 2013; Fuchs and Rochet-Capellan, 2021). According to a broad literature, speech breathing varies not only over time but between individuals (Serre et al., 2021) and the sources of variation are associated not only with cognitive demands, but also with stress, physical, emotional, and motivational aspects (Fuchs and Rochet-Capellan, 2021). As for the representativeness of the findings in a definite period, our data suggest that to some extent the time window matters depending of type of tasks. In our study this feature was found to be preserved on the paced-tasks, that is on reading of single words and object naming. However, it is an open question whether this concept can be applied to tasks such as the VFTs that due to their nature present variable amount of word generation over time. The issues of stability of respiratory demands and representativeness in speaking tasks with different cognitive demands, should be a matter of exclusive investigation in future studies.

### 6.1. Limitations, strengths, and future research

In addition to the limitation already discussed concerning the use of a pneumotachograph mask, other potential limitations exist. In the present study we selected tasks that not only varied in terms of cognitive difficulty but also in regard to the amount of word output. Due to their own nature, the required answers on each task differed from several words produced rapidly, to timed utterances and single words produced freely. The difference on the number of produced words across tasks can be regarded as a limitation, since variation in number of generated words necessarily has an impact on respiration. However, based on existing literature, our selection of tasks stands up as a trade-off in this line of investigation. Already several researchers in linguistics have acknowledged the difficulties in studying speech breathing in free speaking tasks due to the unpredictability of speech production (see Fuchs and Rochet-Capellan, 2021, for a review). An attempt conducted by Mitchell et al. (1996) illustrates this state of affairs. In this study, two free speaking tasks with purportedly different difficulty levels were selected, and respiratory function measured. The results showed no differences in respiratory parameters, and the authors concluded that breathing was not affected by cognitive difficulty. Though, the authors acknowledge that possibly their tasks did not represent different cognitive demands. In contrast to the latter example, we selected tasks differing in cognitive difficulty and word outcome and restricted the statistical analyses to data for the first 15 s. In this way, we were able to discern that the verbal task with the highest cognitive difficulty, VFT, exerted unique changes on breathing. Furthermore, by assessing tasks with different amount of word outcomes, we were able to identify a factor influencing breathing in the experimental set, namely the role of paced word generation. This issue regards reading of single words and object naming, where the stimuli were controlled and time limited. Under such circumstances, no breathing differences appeared between these tasks even if they greatly differ in cognitive requirements. Thus, it is possible that by controlling too much the amount of verbal output, breathing requirements for certain cognitive operations become masked, at least with the instrumentation used in our study. An alternative approach to address the matter is the implementation of tests with serial presentation of stimuli (words or objects), like the “Rapid Automatized Naming” (RAN) task (Wolf et al., 2000). In these types of tasks, several unrelated words/objects are presented simultaneously in series, and subjects need to scan and name the stimuli at their own pace (see Kirby et al., 2010). By evaluating single reading of words and object naming in a serial way, one will get insight into possible respiratory differences between the tasks. Similarly, exploring the effects of procedures where stimuli presentation is self-paced might cast further light into the underlying processes of these tasks.

Because research on how cognitive demands interplay with speech breathing is still largely unknown, the respiratory requirements of other verbal tasks with high cognitive constraints and concrete cognitive operations need to be further assessed. In addition, the role of word length should be explored in future studies and replication of the present data in larger samples and with other languages than Norwegian is recommended. The latter is especially important since Norwegian is one of the most dialect-speaking countries in Europe (Leon, 2014) and our sample of participants was mainly conformed by university students coming from all around the country. Thus, a variation in words’ pronunciation exist. The possible association between pronunciation, cognitive demands and respiration should be addressed in future research. Also, it would be of great interest to expand the present findings by applying different apparatus for acquisition of respiratory parameters and include additional physiological parameters, such as those related to cardiovascular function.

## 7. Conclusion

In spite of the constraints inherent to the experimental study of respiration during speaking, the present investigation showed that execution of a verbal task with high cognitive demands such as VFT, engages unique respiratory adaptations in terms of high air volumes and peak airflows. Notably, VFTs was the only tasks inducing a significant high PEF, which is a striking finding. Since PEF is suggested to be an important index of brain dynamics, the association with VFT points to the possibility that distinct oxygenation demands are in fact related to concrete cognitive operations. Moreover, we were able to corroborate that reading a text passage imposes strong requirements on respiration manly due to the strong physical effort of fast vocalization. Conversely, the respiratory requirements for object naming could not be defined and the matter remains an open question that deserves further scrutiny with additional methodologies. In summary, the present investigation is a small step forward understanding how task difficulty and definite cognitive-linguistic operations affect speech breathing in real time of execution.

## Data availability statement

The dataset of this study is at the time being not available as it is part of a major ongoing project. Data may be available upon request to the PI at the end of data collection. Requests should be sent to the correspondent author (claudia-rodriguez-aranda@uit.no).

## Ethics statement

The studies involving human participants were reviewed and approved by Regional Research Ethics Committee. The patients/participants provided their written informed consent to participate in this study. The person in the picture is one of the authors (Gullsvåg) and she gives her consent to use the picture.

## Author contributions

MG: project administration, recruitment, data collection, analyses revision, and writing. CR-A: conceptualization, analyses, writing, supervision, revision, and funding. All authors contributed to the article and approved the submitted version.

## Funding

This project was supported by the Faculty of Health Sciences, IPS, UiT the Arctic University of Norway.

## Conflict of interest

The authors declare that the research was conducted in the absence of any commercial or financial relationships that could be construed as a potential conflict of interest.

## Publisher’s note

All claims expressed in this article are solely those of the authors and do not necessarily represent those of their affiliated organizations, or those of the publisher, the editors and the reviewers. Any product that may be evaluated in this article, or claim that may be made by its manufacturer, is not guaranteed or endorsed by the publisher.
