# Supplementary material for: Effects of verbal tasks with varying difficulty on real-time respiratory airflow during speech generation in healthy young adults
Source: Front Psychol. 2023 Jun 15;14:1150354. doi: 10.3389/fpsyg.2023.1150354 (PMC10309038; doi:10.3389/fpsyg.2023.1150354)
Supplement: Supplementary file 1 [file Data_Sheet_1.docx]

Supplementary Material

Effects of verbal tasks with varying difficulty on real-time respiratory airflow during speech generation in healthy young adults

Malin Gullsvåg^1^, and Claudia Rodríguez-Aranda^1*^

^1^Department of Psychology, UiT the Arctic University of Norway, Tromsø, Norway

*** Correspondence:** [claudia.rodriguez-aranda@uit.no](mailto:claudia.rodriguez-aranda@uit.no)

# Supplementary Table 1 Means, standard deviations and univariate statistics for respiratory measures by verbal task.

|  | Reading single words | | Reading text passage  (10 words) | | Object  naming | | Semantic fluency | | Phonemic verbal fluency | | *F* (*4, 116*) | *p* | *η_p_^2^* |
| --- | --- | --- | --- | --- | --- | --- | --- | --- | --- | --- | --- | --- | --- |
|  | *M* | *SD* | *M* | *SD* | *M* | *SD* | *M* | *SD* | M | *SD* |  |  |  |
| Insp. Airflow Duration | 5.08 | .98 | 0.95 | .51 | 5.12 | .80 | 4.35 | .90 | 4.62 .89 | | 165.12 | .001 | .85 |
| Exp. Airflow Duration | 9.90 | .98 | 3.63 | .77 | 9.85 | .80 | 10.57 | .89 | 10.33 .89 | | 460.85 | .001 | .94 |
| Peak Insp. Airflow | -.89 | .32 | -1.29 | .49 | -.90 | .39 | -1.47 | .44 | -1.41 .45 | | 16.54 | .001 | .36 |
| Peak Exp. Airflow | .65 | .23 | .59 | .27 | .67 | .28 | 1.00 | .50 | 1.06 .58 | | 15.52 | .001 | .35 |
| Insp. Volume | -1.83 | .65 | -.57 | .26 | -1.78 | .57 | -2.22 | .72 | -2.36 .77 | | 55.96 | .001 | .66 |
| Exp. Volume | 1.97 | .58 | .59 | .22 | 1.97 | .71 | 1.91 | .64 | 2.02 .87 | | 44.01 | .001 | .60 |

**Supplementary Table 2.** Means, standard deviations and univariate statistics for respiratory measures by verbal task for first 30 seconds.

|  | Reading single words | | Reading text passage | | Object Naming | | Semantic Fluency | | Phonemic Fluency | | *F* (*4,116*) | *p-value* | *η_p_^2^* |
| --- | --- | --- | --- | --- | --- | --- | --- | --- | --- | --- | --- | --- | --- |
|  | *M* | *SD* | *M* | *SD* | *M* | *SD* | *M* | *SD* | *M* | *SD* |  |  |  |
| Insp. Airflow Duration | 10.40 | 1.33 | 4.13 | 1.20 | 11.25 | 1.88 | 8.62 | 1.63 | 9.13 | 1.45 | 145.01 | *p* < .001 | .83 |
| Exp. Airflow Duration | 19.55 | 1.32 | 22.16 | 2.31 | 18.69 | 1.88 | 21.32 | 1.64 | 20.81 | 1.44 | 24.63 | *p* < .001 | .46 |
| Peak Insp. Airflow | -1.01 | .36 | -2.31 | .74 | -1.05 | .51 | -1.59 | .49 | -1.52 | .57 | 51.11 | *p* < .001 | .64 |
| Peak Exp. Airflow | .77 | .29 | .79 | .35 | .79 | .35 | 1.25 | .57 | 1.31 | .758 | 17.13 | *p* < .001 | .37 |
| Insp. Volume | -4.07 | 1.19 | -2.69 | .94 | -3.96 | 1.24 | -4.35 | 1.24 | -4.34 | 1.43 | 16.72 | *p* < .001 | .37 |
| Exp. Volume | 4.20 | 1.20 | 3.00 | .96 | 3.88 | 1.19 | 4.15 | 1.35 | 4.22 | 1.68 | 8.45 | *p* < .001 | .23 |

## Supplementary Figures

**Figure A**
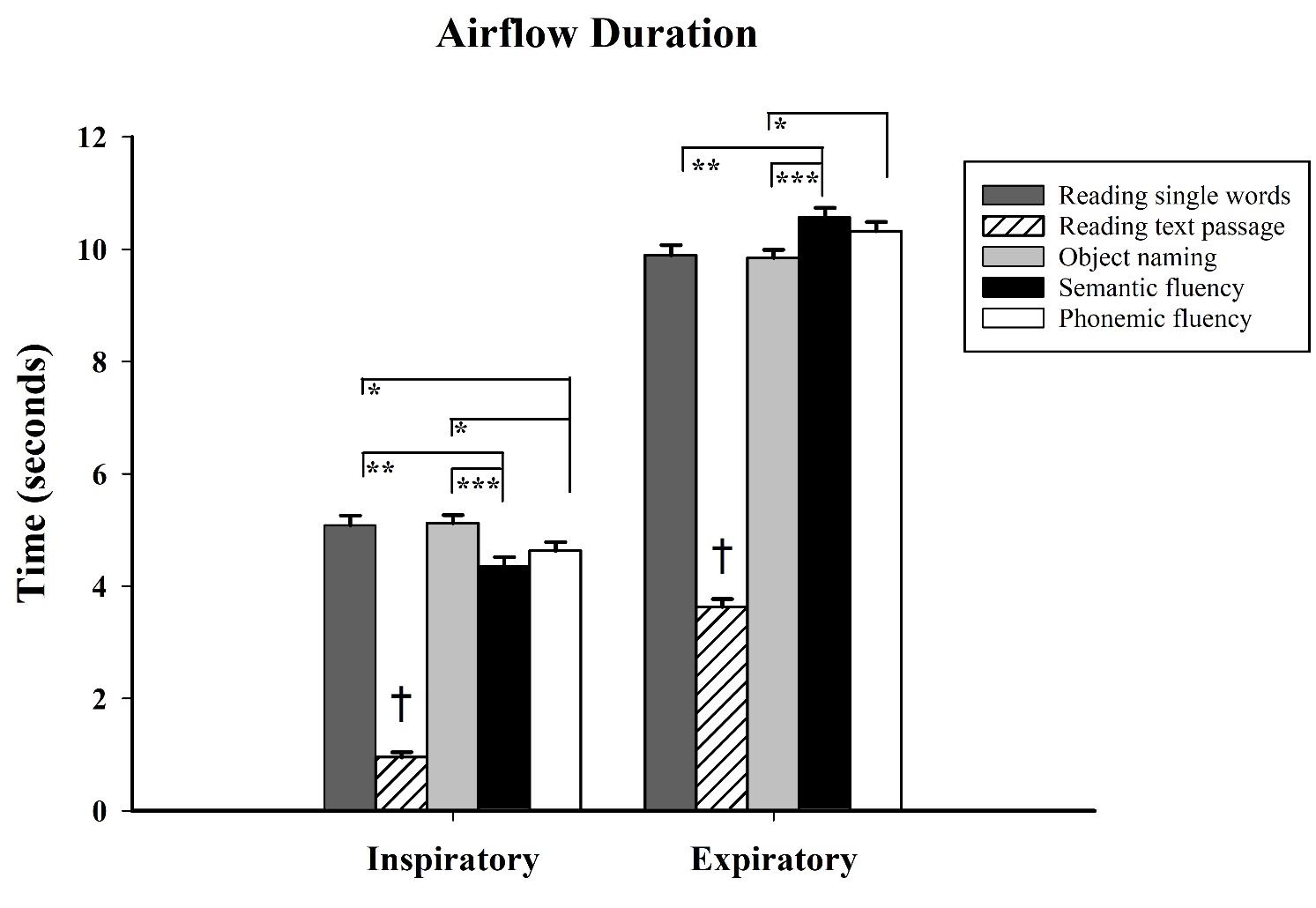


**Figure B**


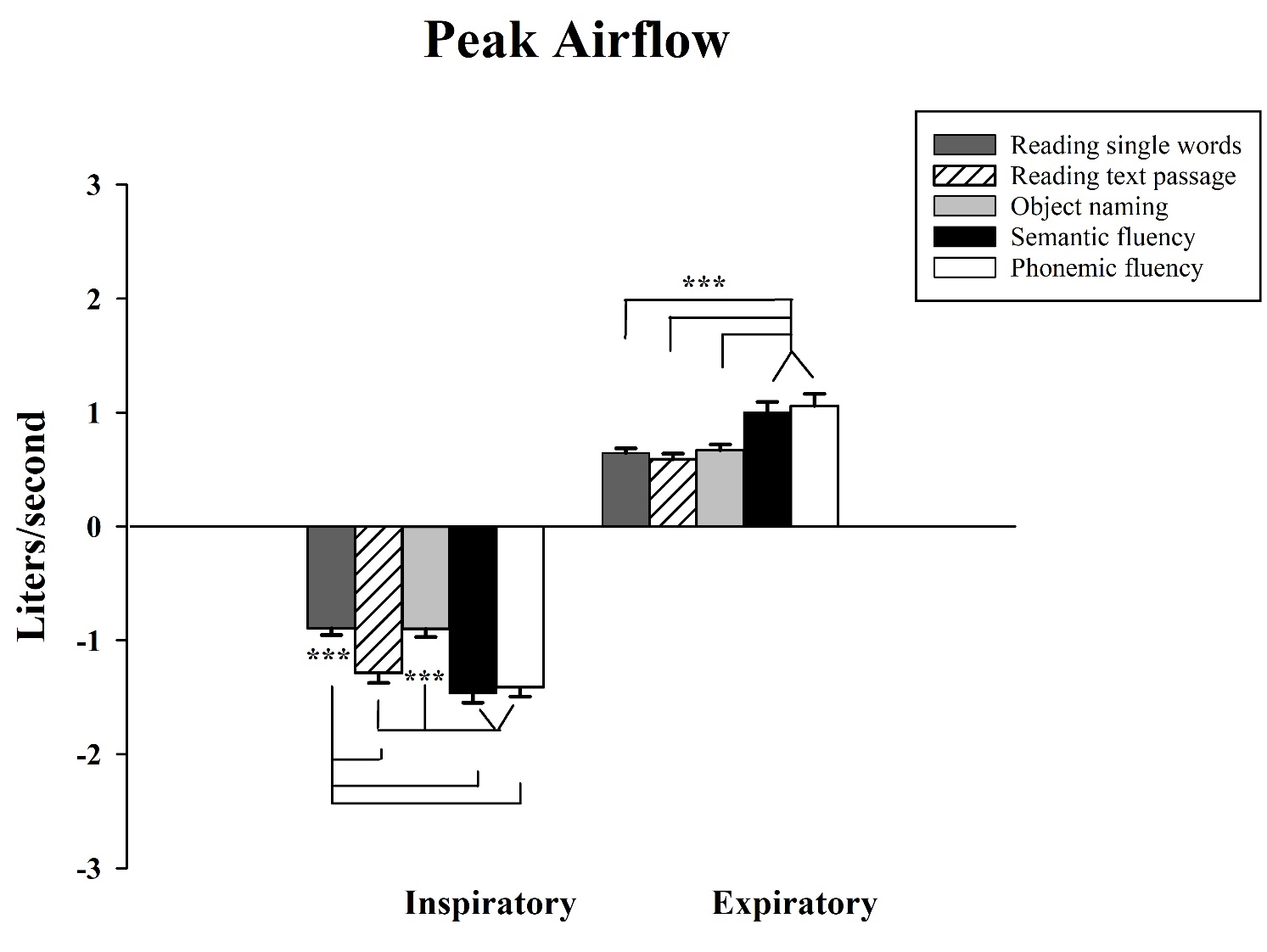


**Figure C**


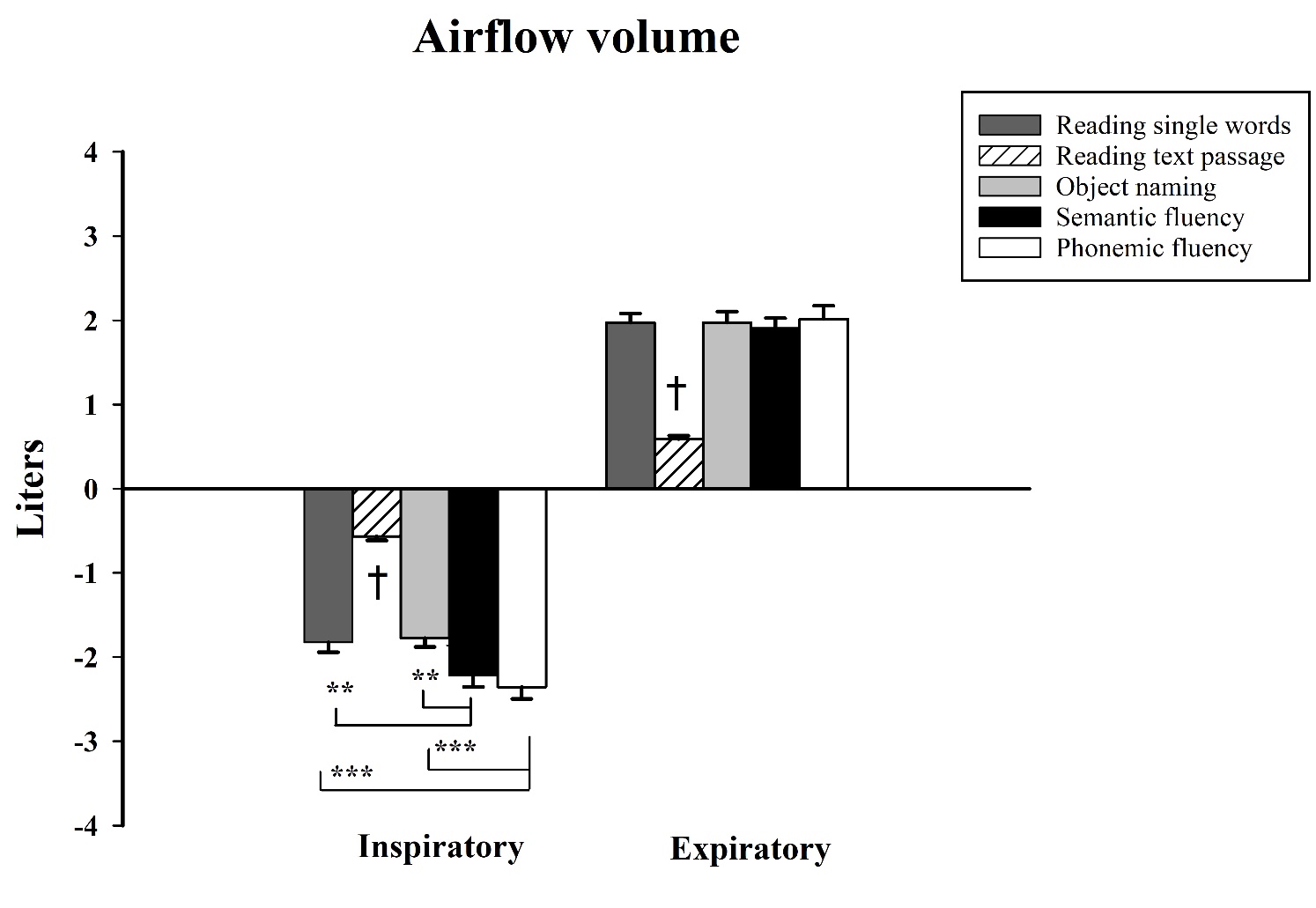


**Figure Captions**

**Supplementary Figure A.** Mean ± *SEM* for airflow duration measured in seconds. Verbal tasks shown on horizontal axis. † = different from all tasks; *** < .001, ** < .01, * < .05. Measurements were conducted for adjusted number of words for “reading a text passage”. Ten words from this test were used and contrasted against results in reading single words, object naming, semantic fluency and phonemic fluency for first 15 seconds.

**Supplementary Figure B.** Mean ± *SEM* for peak airflow measured in liters per second. Verbal tasks shown on horizontal axis. *** < .001, ** < .01, * < .05. Measurements were conducted for adjusted number of words for “reading a text passage”. Ten words from this test were used and contrasted against results in reading single words, object naming, semantic fluency and phonemic fluency for first 15 seconds.

**Supplementary Figure B.** Mean ± *SEM* for airflow volume measured in liters. Verbal tasks shown on horizontal axis. † = different from all tasks; *** < .001, ** < .01, * < .05. Measurements were conducted for adjusted number of words for “reading a text passage”. Ten words from this test were used and contrasted against results in reading single words, object naming, semantic fluency and phonemic fluency for first 15 seconds.
